# Tumor Necrosis Factor Alpha Inhibition for Inflammatory Bowel Disease after Liver Transplant for Primary Sclerosing Cholangitis

**DOI:** 10.1155/2018/1015408

**Published:** 2018-05-15

**Authors:** Ravish Parekh, Ahmed Abdulhamid, Sheri Trudeau, Nirmal Kaur

**Affiliations:** ^1^Department of Gastroenterology and Hepatology, Henry Ford Health System, Detroit, MI, USA; ^2^Department of Public Health Sciences, Henry Ford Health System, Detroit, MI, USA

## Abstract

**Background:**

Outcome data regarding the use of tumor necrosis factor alpha inhibitors (anti-TNF*α*) in patients with inflammatory bowel disease (IBD) after liver transplant (LT) for primary sclerosing cholangitis (PSC) are scant.

**Methods:**

We performed a retrospective chart review to investigate outcomes among a series of post-liver-transplant PSC/IBD patients receiving anti-TNF*α* therapy at Henry Ford Health System ((HFHS), Detroit, MI).

**Results:**

A total of five patients were treated with anti-TNF*α* agents for IBD after LT for PSC from 1993 through 2015. Two patients were treated with adalimumab, and three were treated with infliximab. Three patients were hospitalized with severe posttransplant infections. Two patients developed posttransplant lymphoproliferative disease (PTLD); one of these patients died due to complications of PTLD.

**Conclusion:**

Anti-TNF*α* treatment following LT worsened the disease course in our patients with concurrent PSC/IBD and led to serious complications and surgical intervention. Larger studies are needed to evaluate the side effects and outcomes of the use of such agents in this patient population. Until then, clinicians should have a high threshold to use anti-TNF*α* therapy in this setting.

## 1. Introduction

The co-occurrence of inflammatory bowel disease (IBD) and primary sclerosing cholangitis (PSC) is a well-documented phenomenon. Although there are no epidemiological studies regarding the prevalence of concurrent PSC/IBD, as many as 90% of patients with PSC may have underlying IBD [1, 2]. No medical therapy has yet been proven to affect the natural progression of PSC and therefore, liver transplant (LT) remains the mainstay of therapy for patients with advanced cirrhosis secondary to the disease; without transplant, the mean survival of patients with PSC is 10–12 years [3–5]. Compared to patients with IBD alone, patients with cooccurring PSC/IBD generally present with a different clinical course, mainly characterized by a high prevalence of pancolitis with rectal sparing and backwash ileitis [6].

In recent years, multiple agents have been approved for the treatment of IBD. However, tumor necrosis factor alpha inhibitors (anti-TNF*α*) remain widely used, given their widespread demonstrated efficacy in moderate, severe, and refractory IBD [7, 8]. However, the risks and benefits of these agents in PSC/IBD patients after liver transplant are yet to be determined. We examined the clinical course of PSC/IBD patients receiving liver transplants and treated with anti-TNF*α* agents.

## 2. Methods

This study was approved by the HFHS Institutional Review Board; requirements for written informed consent were waived due to the deidentified nature of the study. A retrospective chart review of our patient database was performed, using International Classification of Diseases, version 9 (ICD-9) codes related to Crohn's disease (555.0, 555.1, 555.9), ulcerative colitis (556.9), PSC (576.1), and LT (V42.7). Using this method, we identified five patients with concurrent PSC/IBD who underwent liver transplantation and also received anti-TNF*α* therapy at HFHS between 1993 and 2015. Three trained gastroenterologists (RP, AAH, and NK) performed retrospective chart review for data including demographic data (sex, age, and race); hospital admissions (indications); medical treatment, including prednisone escalation for IBD; endoscopy results; surgery; and infectious complications. The aim of the study was to assess the clinical effectiveness (defined as the absence of symptoms and endoscopic remission) and safety of biologic therapy in this clinical scenario.

## 3. Results

A total of five post-LT PSC/IBD patients were treated with anti-TNF*α* agents from 1993 through 2015 at HFHS. Two patients were treated with adalimumab, and three were treated with infliximab. See summary results in Table 1.

### 3.1. Subject 1

A 9-year-old white male was diagnosed with pancolonic Crohn's disease and responded well to treatment with prednisone, azathioprine, and then methotrexate. Two years following his IBD diagnosis, the patient was found to have primary sclerosing cholangitis with bridging fibrosis and cirrhosis. At age 17, the patient received a deceased donor liver transplant, after which he received mycophenolate mofetil and cyclosporine for immunosuppression. Following the transplant, the patient underwent multiple hospitalizations for cholangitis, perihepatic abscess secondary to methicillin-resistant Staphylococcus aureus (MRSA), cytomegalovirus (CMV) viremia, cellulitis, and esophageal candidiasis. The patient's posttransplant course was also complicated by worsening Crohn's colitis, treated with adalimumab. Despite this therapy, however, his colitis continued to worsen. He subsequently developed toxic megacolon and underwent a subtotal abdominal colectomy with end-ileostomy for refractory colitis. For approximately 5 months following his colectomy, the patient had marked improvement in pain, appetite, and functional status. He subsequently began to develop strictures at the ileostomy site secondary to active colitis with small bowel involvement, requiring multiple office visits for dilation of the ileostomy site. The patient was eventually hospitalized with worsening abdominal pain. Endoscopic ultrasound with biopsy showed malignant lymphoma, consistent with posttransplant lymphoproliferative disease (PTLD). Despite treatment with rituximab and corticosteroids, the patient continued to decompensate and eventually expired due to complications of PTLD.

### 3.2. Subject 2

A white female patient was diagnosed with ulcerative colitis at age 18, and PSC at age 20. The patient's colitis symptoms were initially well-controlled with azathioprine and mesalamine. At age 25, the patient received a living-donor liver transplant subsequent to PSC; posttransplant immunosuppressant treatment included tacrolimus and azathioprine in addition to mesalamine. Following transplant, the patient experienced symptoms of worsening colitis, with frequent flare-ups requiring multiple courses of high-dose prednisone for disease control. Despite the steroid treatments, the patient's symptoms continued to worsen. Subsequent treatment with infliximab resulted in marked improvement in her symptoms. However, the patient's course was complicated by pancytopenia. She continued to have active IBD symptoms following her transplant.

### 3.3. Subject 3

A white male patient was diagnosed with ulcerative colitis at age 29 and responded well to ASA and azathioprine therapy. He was diagnosed with PSC at age 32 and received a deceased donor liver transplant at age 41. He had a history of recurrent clostridium difficile infections requiring fecal transplantation prior to the transplant. Following liver transplant, the patient was hospitalized for MRSA bacteremia, pneumonia with severe sepsis, recurrent MRSA pneumonia, and recurrent clostridium difficile infections. However, despite treatment with azathioprine, his colitis symptoms began to worsen posttransplant and required escalation of prednisone dose. Azathioprine was subsequently discontinued and infliximab started in response to worsening colitis. Prednisone therapy was tapered off due to side effects. At of the end of follow-up, the patient was maintained on infliximab and budesonide; his UC was in clinical remission.

### 3.4. Subject 4

A white female patient was diagnosed with ulcerative colitis at age 26 and PSC at age 33. Prior to transplant, the patient was maintained on mesalamine and her UC was under good control, with no evidence of active colitis on colonoscopy. The patient's PSC was initially asymptomatic but quickly deteriorated, with multiple hospital admissions for episodes of cholangitis. At age 48, she received a liver transplant from a deceased donor for end-stage liver disease secondary to PSC. Posttransplant, the patient was started on mycophenolate mofetil, tacrolimus, and prednisone for immunosuppression. Her course was complicated by acute cellular rejection, which was treated with an increased dose of corticosteroids. The patient also experienced worsening of colitis. Mesalamine therapy was reinitiated with poor response; treatment was changed to infliximab, but symptoms continued to worsen. Her course was further complicated by clostridium difficile colitis, which did not respond to antibiotics and subsequently required a fecal transplant. Due to uncontrolled colitis with worsening symptoms, the patient underwent a colectomy with ileostomy, with marked improvement in her symptoms and overall health following surgery.

### 3.5. Subject 5

A white male patient was diagnosed with Crohn's disease at age 29 and PSC at age 33. Prior to liver transplant, the patient's Crohn's disease was in remission, maintained on adalimumab. Following transplant of a deceased donor liver at age 45, the patient was continued on adalimumab, with tacrolimus added for immunosuppression. His posttransplant course was complicating by posttransplant lymphoproliferative disease (PTLD); 9 months after transplant, adalimumab was discontinued. The patient was started on cyclophosphamide for his PTLD with interval improvement in disease activity on his most recent PET scan. The patient's Crohn's disease symptoms remained under good control, with multiple colonoscopies showing no evidence of flare-ups of his disease. However, the patient's course was further complicated by acute cellular rejection and recurrence of PTLD. At last follow-up, the patient was maintained on mycophenolate, tacrolimus, and prednisone therapy, in addition to cyclophosphamide for PTLD. The addition of mesalamine has provided only partial relief from symptoms.

## 4. Discussion

Our patient experience suggests that anti-TNF*α* agents appear to be both relatively unsafe for patients with IBD after liver transplant and less effective at mitigating the disease than in patients without liver disease or transplant. Two patients went on to require a colectomy for severe colitis with immediate improvement in symptoms following the surgery. While our patients did well after colectomy, undergoing such a major operation in the post-LT setting is a high-risk scenario that should ideally be avoided. These outcomes demonstrate that these anti-TNF*α* agents can be poorly effective in the post-LT setting, in stark contrast to the known effectiveness of these therapies in patients without transplant.

Our study also demonstrates the severity of anti-TNF*α*-related complications in the post-LT setting. After transplant, three of five patients treated with anti-TNF*α* agents developed serious infections, including clostridium difficile colitis, esophageal candidiasis, CMV viremia, MRSA bacteremia, and community acquired pneumonia requiring multiple hospitalizations. In addition, two patients developed PTLD while being treated with an anti-TNF*α* agent, and one patient died due to this condition. This relatively high rate of such severe and potentially fatal complications is disproportionate to what is generally observed with anti-TNF*α* agents and suggests an underlying pathophysiology that is specific to the post-LT setting.

A previous study (*n* = 8) [9] of anti-TNF*α* agents in PSC/IBD patients reported similar outcomes. Four patients developed opportunistic infections (esophageal candidiasis, Clostridium difficile colitis, community acquired bacterial pneumonia, and cryptosporidiosis); one patient developed PTLD. This is consistent with our own observations; it is possible that anti-TNF*α* agents increase risk of PTLD among these patients. In contrast, however, that study also observed improvement in IBD-related clinical outcomes as well as mucosal healing. Another similar study (*n* = 6) [10] described significant improvement in IBD-related symptoms in four patients following the use of infliximab therapy.

Our case series is limited by the small number of patients observed; although this is a reflection of the relative rarity of IBD/PSC-LT in the population, we are hesitant to generalize the results to a whole population. Furthermore, given the variation in both the IBD subtype (Crohn's disease versus ulcerative colitis), the timing and type of anti-TNF*α* agents that each patient received, and the posttransplant immunosuppressive regimens used, it is difficult to isolate the effects of the anti-TNF*α* treatment [11] on disease activity. In particular, it is important to note that tacrolimus and immunosuppressive medications may also contribute to the risk of adverse clinical outcomes, especially infections, observed among these patients [12].

In summary, this case report illustrates that—despite widespread use of anti-TNF*α* agents in patients with refractory IBD—clinicians should exercise caution when employing these medications in the treatment of patients after liver transplant. Given the potential for significant complications, choice of immunosuppressive therapy and IBD treatment should be carefully considered; patients should be counseled regarding the possibility of an IBD exacerbation prior to transplant and monitored closely afterward. Further, large-scale studies are needed to evaluate the safety and efficacy of anti-TNF*α* therapies in IBD/PSC patients.

## Figures and Tables

**Table 1:** Five patients with inflammatory bowel disease, primary sclerosing cholangitis, and liver transplant treated with antitumor necrosis factor alpha agents.

	IBD type	Age at IBD onset	Age at PSC onset	Age at LT	Pre-LT TNF*α* agent	Liver donor status	Post-LT TNF*α* agent	Concomitant immunosuppression	Complications	IBD disease activity
Patient 1: white male	Crohn's	9	11	17	None	Deceased	Adalimumab	Cyclosporine; mycophenolate mofetil	C. diff colitis, esophageal candidiasis, CMV viremia, PTLD, Death	Hospital admission; prednisone escalation; colectomy

Patient 2: white female	UC	18	20	25	None	Living unrelated	Infliximab	Tacrolimus; azathioprine	Pancytopenia	Hospital admission; prednisone escalation; active colitis

Patient 3: white male	UC	29	32	41	None	Deceased	Infliximab	Tacrolimus	MRSA bacteremia; pneumonia with sepsis; C. diff. colitis.	Hospital admission; prednisone escalation; active colitis

Patient 4: white female	UC	26	33	48	None	Deceased	Infliximab	Tacrolimus; mycophenolate mofetil	Acute rejection; C. diff colitis	Hospital admission; colectomy

Patient 5: white male	Crohn's	29	33	45	Adalimumab	Deceased	Adalimumab	Tacrolimus	PTLD	Active colitis

IBD: inflammatory bowel disease; PSC: primary sclerosing cholangitis; LT: liver transplant; TNF*α*: tumor necrosis factor alpha; C. diff: Clostridium difficile; CMV: cytomegalovirus; PTLD: posttransplant lymphoproliferative disease; UC: ulcerative colitis.
